# Potential role of multiple carbon fixation pathways during lipid accumulation in *Phaeodactylum tricornutum*

**DOI:** 10.1186/1754-6834-5-40

**Published:** 2012-06-06

**Authors:** Jacob Valenzuela, Aurelien Mazurie, Ross P Carlson, Robin Gerlach, Keith E Cooksey, Brent M Peyton, Matthew W Fields

**Affiliations:** 1 Department of Biochemistry and Chemistry, Bozeman, USA; 2 Center for Biofilm Engineering, Bozeman, USA; 3 Department of Microbiology, Bozeman, USA; 4 Bioinformatics Core, Bozeman, USA; 5 Department of Chemical and Biological Engineering, Montana State University, Bozeman, MT, 59717, USA; 6 Center for Biofilm Engineering, 366 EPS Building, Montana State University, Bozeman, MT, 59717, USA

**Keywords:** Algae, Diatom, Lipid-accumulation, Transcriptomics, Biofuel, Carbon fixation, RNA-seq, Bio-oil

## Abstract

**Background:**

*Phaeodactylum tricornutum* is a unicellular diatom in the class *Bacillariophyceae.* The full genome has been sequenced (<30 Mb), and approximately 20 to 30% triacylglyceride (TAG) accumulation on a dry cell basis has been reported under different growth conditions. To elucidate *P. tricornutum* gene expression profiles during nutrient-deprivation and lipid-accumulation, cell cultures were grown with a nitrate to phosphate ratio of 20:1 (N:P) and whole-genome transcripts were monitored over time via RNA-sequence determination.

**Results:**

The specific Nile Red (NR) fluorescence (NR fluorescence per cell) increased over time; however, the increase in NR fluorescence was initiated before external nitrate was completely exhausted. Exogenous phosphate was depleted before nitrate, and these results indicated that the depletion of exogenous phosphate might be an early trigger for lipid accumulation that is magnified upon nitrate depletion. As expected, many of the genes associated with nitrate and phosphate utilization were up-expressed. The diatom-specific cyclins *cyc*7 and *cyc*10 were down-expressed during the nutrient-deplete state, and cyclin B1 was up-expressed during lipid-accumulation after growth cessation. While many of the genes associated with the C3 pathway for photosynthetic carbon reduction were not significantly altered, genes involved in a putative C4 pathway for photosynthetic carbon assimilation were up-expressed as the cells depleted nitrate, phosphate, and exogenous dissolved inorganic carbon (DIC) levels. *P. tricornutum* has multiple, putative carbonic anhydrases, but only two were significantly up-expressed (2-fold and 4-fold) at the last time point when exogenous DIC levels had increased after the cessation of growth. Alternative pathways that could utilize HCO_3_^-^ were also suggested by the gene expression profiles (*e.g*., putative propionyl-CoA and methylmalonyl-CoA decarboxylases).

**Conclusions:**

The results indicate that *P. tricornutum* continued carbon dioxide reduction when population growth was arrested and different carbon-concentrating mechanisms were used dependent upon exogenous DIC levels. Based upon overall low gene expression levels for fatty acid synthesis, the results also suggest that the build-up of precursors to the acetyl-CoA carboxylases may play a more significant role in TAG synthesis rather than the actual enzyme levels of acetyl-CoA carboxylases *per se*. The presented insights into the types and timing of cellular responses to inorganic carbon will help maximize photoautotrophic carbon flow to lipid accumulation.

## Background

Since the industrial revolution, the infrastructure of our society has relied strongly on petroleum-based products for fuels, materials, and specialty chemicals. For the last hundred years, the use of petroleum has been possible due to a balance between supply and demand. However, the increased consumption of energy has created an environment where need could begin to exceed supplies [1]. Perhaps even more important are the environmental impacts of petroleum consumption. In terms of carbon, the fossil fuels consumed in one year release 44 × 10^18^ g of carbon, and this is 400-fold the amount of annual carbon fixed during net primary productivity by the global biota [2]. This is a massive influx of carbon into the atmosphere mediated through the burning and consumption of petroleum-based products, and it is becoming increasingly clear that renewable biofuels (*e.g.*, ethanol, butanol, H_2_, CH_4_, biodiesel) are needed to help replace petroleum-dependence in the United States and the world. However, while present technology can be used to help circumvent and reverse current environmental trends, both fundamental and applied research is needed to advance the feasibility and utility of renewable energy sources that use direct phototrophic CO_2_-fixation into liquid biofuels.

Diatoms are a diverse group of eukaryotic unicellular microalgae that account for up to 40% of the total marine primary production each year [3-5]. In addition to photoautotrophic growth (*i.e.*, carbon fixation via sunlight), some green algae and diatoms can store carbon and energy in the form of lipids (*i.e.*, triacylglycerides, TAGs), and this fact has re-invigorated the possibilities of algal oil being used for the production of liquid fuels. Chisti (2007) estimates that biodiesel from microalgae would only take 3% of the arable crop land in the U.S. to replace 50% of the country’s liquid transportation fuel needs [6]. Courchensne et. al. 2009 [7] reviewed many of the efforts focused on increasing microalgal lipid production, including biogeochemical and genetic approaches. Although we understand many aspects of carbon assimilation in diatoms, the direct responses and contributive flow of inorganic carbon to lipid accumulation are not known for many eukaryotic photoautotrophs under different growth conditions [8]. Most approaches have had some success, but there is still much work to be done to fully understand the efficient and economical enhancement of lipid production in microalgae.

The marine diatom, *Phaeodactylum tricornutum,* is classified in the phylum *Bacillariophyta*, and this phylum comprises one-third of all known marine phytoplankton. *P. tricornutum* is a chlorophyll c-containing alga known as a heterokont [9], and has been studied as a ‘model’ diatom in the context of physiology, biochemistry, and genomics. *P. tricornutum* is a pleomorphic diatom that has been isolated and classified into 10 different strains over the last century based upon genetic and phenotypic differences [10]. *P. tricornutum* 8.6 (CCAP 1055/1; CCMP2561; strain Pt1) has a major morphotype of fusiform and was selected for whole genome sequence determination. The Pt1 strain has a 27.4 Mb genome with over 10,000 predicted genes [11], and the chloroplast genome sequence has also been determined (117,000 bp; 162 genes) [12]. This wealth of genomic knowledge has revealed the evolutionary lineage of diatoms and has also uncovered the physiological potential of lipid-accumulating diatoms and green algae. Accompanying the sequenced genomes are over 130,000 ESTs (Expressed Sequence Tags) from 16 different growth conditions of *Phaeodactylum tricornutum*[13], and the data is compiled in the Diatom EST Database [14].

This extensive research background is the foundation for using Pt1 as a model diatom system to better understand cellular responses during lipid-accumulation under different growth conditions. Nutrient deficiency or nutrient stress has been well documented to increase TAG accumulation in microalgae [15]. Specifically, nitrogen- or phosphate-limitation can increase lipid accumulation in numerous microalgae [16], and TAG accumulation in *Phaeodactylum* has been studied under nitrogen depletion [16]. In the current study we used the model diatom, *P. tricornutum* strain Pt1, to characterize global gene expression via RNA-seq during enhanced lipid production as a consequence of nitrogen- and phosphate-depletion.

## Results and discussion

### Depletion of nitrate and phosphate

**Figure 1:**
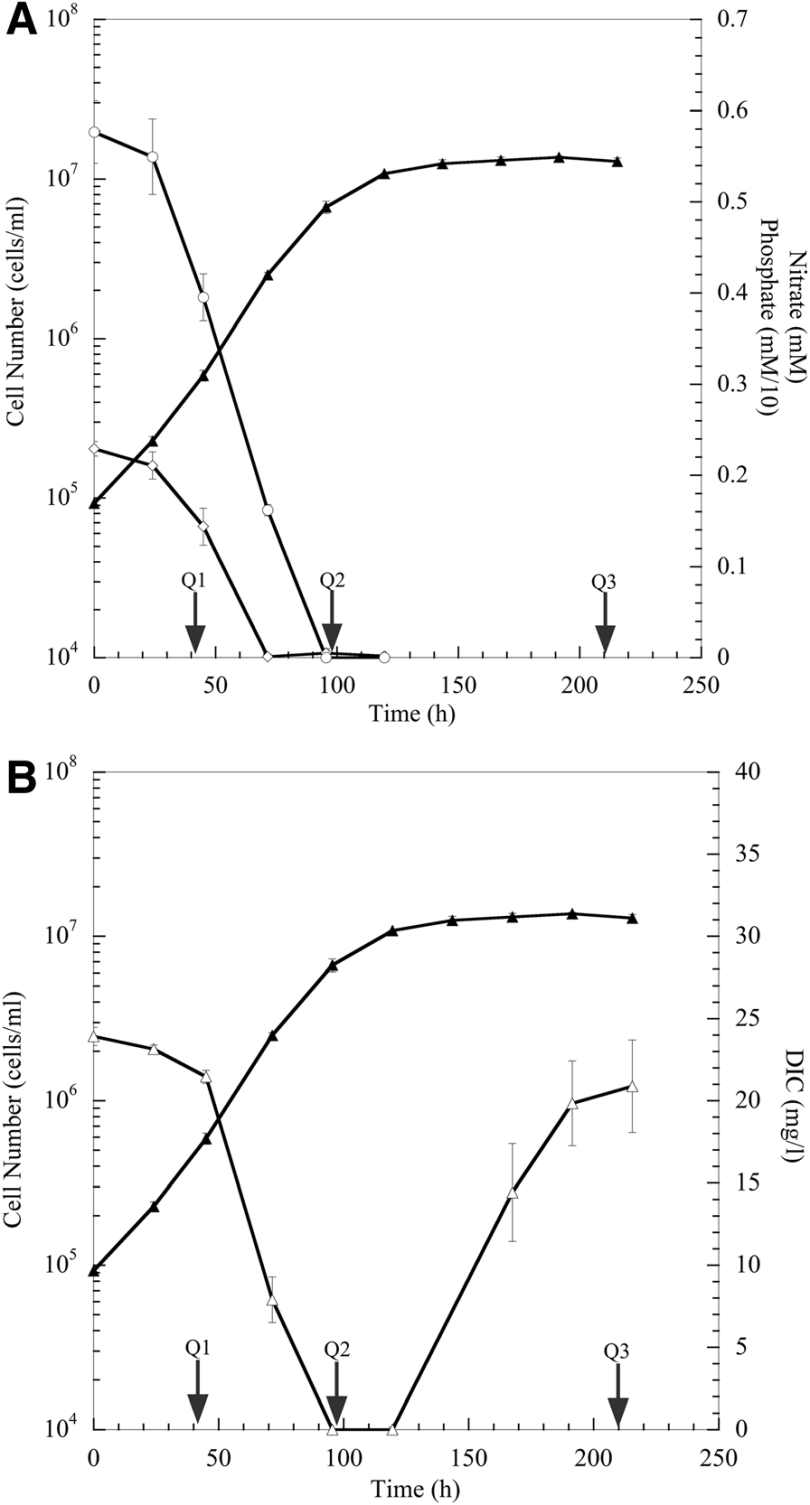
**Growth characterization of*P. tricornutum***. Cell density growth curve of *P. tricornutum* cells (▲) showing depletion of exogenous nitrate (○) and phosphate (◊). Phosphate concentrations are multiplied by a factor of 10 for visualization **(A)**. Cell density growth curve showing the depletion and rebound of dissolved inorganic carbon (∆) throughout *P. tricornutum* growth **(B)**. Arrows indicate time points at which cells were harvested for RNA sequencing analysis.

Sodium nitrate and potassium phosphate were the only sources of nitrogen or phosphorus available during growth of *P. tricornutum,* and ASPII medium was used as recently described [17]. The exogenous nitrate and phosphate was monitored daily to determine nutrient availability. The classic Redfield ratio of nitrogen/phosphorus (N/P) is 16:1 [18,19] in phytoplankton, however it can be dependent on the source of nitrogen [20]. The growth medium in the described experiments had a N:P ratio of approximately 20.5:1 [21,22]. Growth parameters and gene expression were measured at three time points, early-exponential (Q1), transition from exponential to stationary-phase (Q2), and stationary-phase (Q3). The growth data (Figure 1a) suggested that nitrogen and not phosphate depletion coincided with the onset of stationary-phase. Exogenous phosphate was depleted after 72 h, but exponential growth continued for another 24 h. At 96 h, the exogenous nitrate was depleted and cells transitioned to stationary-phase within one doubling-period. At this time, a decrease in chlorophyll a content was observed ( Additional file 1: Figure S1), and these results suggest a recycling of nitrogen rich compounds (*e.g*., chlorophyll). Similar results have been observed during nitrogen depletion in the green alga, *Neochloris oleoabundans*[23]. Elemental analysis ( Additional file 2: Table S1) revealed that cells in nutrient-replete Q1 had a N:P ratio of 4:1, but with the depletion of both exogenous nitrate and phosphate, cellular N:P ratios shifted to 8:1 and 9:1 in Q2 and Q3, respectively. These results indicate that under the tested growth conditions of a starting exogenous N:P ratio of 20.5:1, cells transitioned to stationary-phase at an approximate cellular N:P ratio of 8 to 9.

The availability of dissolved inorganic carbon (DIC) during the light period was high in early-exponential growth, but decreased below detectable levels during the late-exponential phase (Q2) (Figure 1b). Exogenous DIC declined during the exponential growth phase, and the decline continued past the depletion of exogenous nitrate and phosphate (Figure 1b). The decline in DIC followed cell accumulation during exponential-growth. At the onset of nitrate depletion, the light-phase DIC remained low for approximately 25 h, and these results suggested the culture consumed the DIC at the mass transfer rate from the air-sparge. The DIC began to increase when the cells entered stationary-phase. The DIC levels at Q3 (approximately 50 h after the depletion of nitrate) increased back to similar levels observed at the initiation of growth (Figure 1). These results indicate lower carbon fixation under nutrient-deprivation and that biological activity was no longer limited by DIC mass transfer.

### Lipid-accumulation under nutrient stress

**Figure 2:**
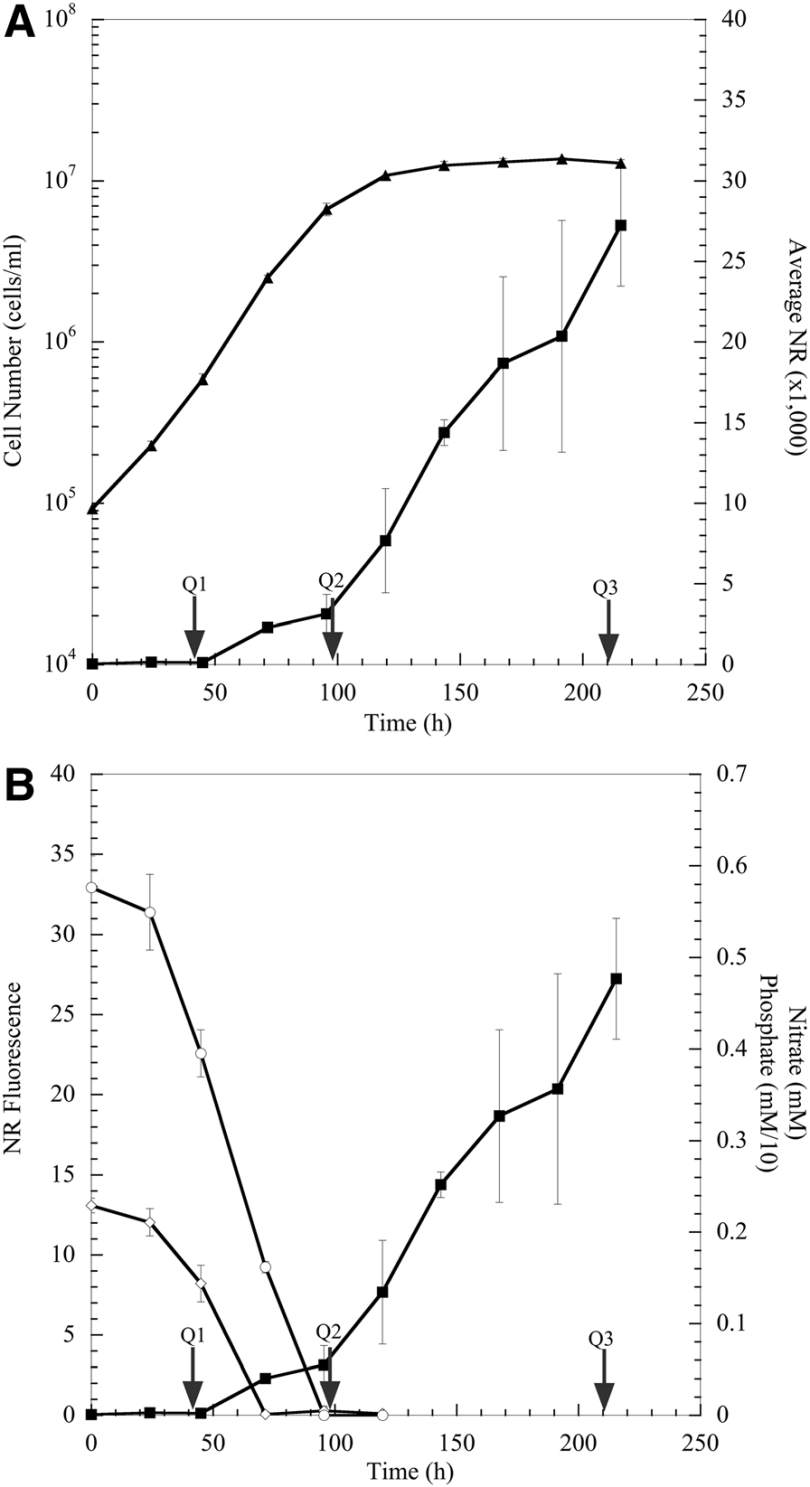
**Characterization of lipid accumulation in*P. tricornutum*during increase in Nile Red fluorescence intensity (■) with respect to cell number (▲) (A).** Nile Red fluorescence intensity indicating the increase in lipids is shown with the depletion of external nitrate (○) and phosphate (**◊**) **(B)**. Phosphate concentrations are multiplied by a factor of 10 for scaling purposes (e.g., 0.2 mM = 0.02 mM). Arrows indicate time points at which cells were harvested for RNA sequencing.

**Figure 3:**
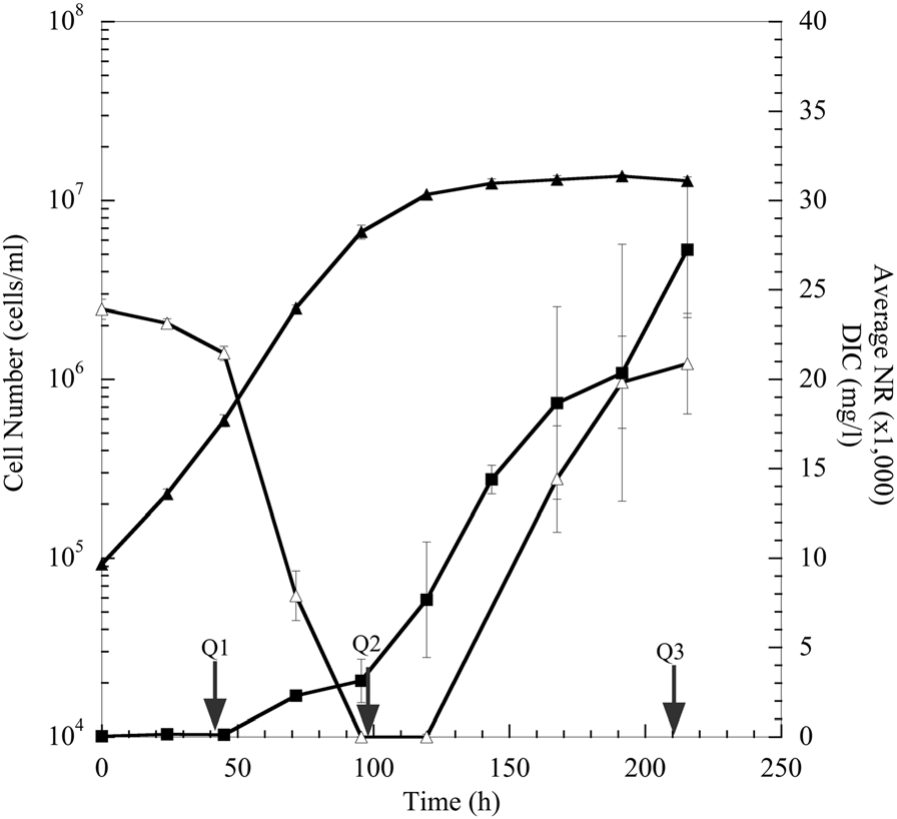
**Nutrient depletion and cell count growth curve of*P. tricornutum.*** Cell density (▲) during the depletion and rebounding of dissolved inorganic carbon (∆) and increase in Nile Red fluorescence intensity (∎). Arrows indicate time points at which cells were harvested for RNA sequencing.

Throughout *P. tricornutum* growth, lipid accumulation was monitored via the Nile Red (NR) assay as a way to measure relative abundance of triacylglycerols [24]. The NR fluorescent intensities increased significantly as the cells transitioned to stationary-phase (approximately 95 h under the tested growth conditions; Figure 2). The specific NR fluorescence (NR fluorescence intensity/cell) continued to increase after 96 h and these results indicate that the lipid accumulation was not merely a result of increasing cell numbers. After nutrient depletion, the NR specific fluorescence increased 4.5-fold (Figure 2a), and the increased fluorescence coincided with a cessation of population growth (Figure 2a). The data indicate that lipids started to increase as exogenous phosphate was depleted, but the rate (specific NR fluorescence/time) increased upon nitrate depletion (Figures 2b and 3). A previous study reported that phosphate limitation could increase lipid content in *P. tricornutum,* but not green flagellates [25]. Our results suggest that depletion of exogenous phosphate might be an early trigger for lipid accumulation that is magnified upon nitrate depletion; and therefore, the N:P ratio could be an important parameter to monitor when examining mechanisms of lipid accumulation.

The lipid accumulation increased with cell numbers as exogenous DIC concentrations decreased, whereas specific lipid accumulation (NR/cell) increased with subsequent increases in exogenous DIC as biomass accumulation ceased (Figure 3). This increase in lipid accumulation could also be characterized in context of the increased cellular carbon to nitrogen ratio (C:N). At Q1 and Q2, the C:N ratio was 7.5:1 and 6.5:1, respectively, which is similar to the Redfield C:N ratio of 6.6. However, as lipids accumulated, the C:N ratio increased to 15.4:1, and previous studies have documented a similar increase in the C:N ratio after exogenous nitrate depletion [26]. Thus, elemental analysis indicated continued carbon influx after exogenous N and P depletion. In addition, specific cellular carbohydrate (μg carbohydrate/cell) did not increase ( Additional file 3: Figure S2), and intracellular lipid droplets were observed via epifluorescent microscopy ( Additional file 4: Figure S3).

### Analysis of RNA-sequence data

A time course assessment strategy was employed to identify transcripts that were differentially expressed during growth and lipid accumulation from early-exponential (Q1), late-exponential (Q2), and stationary-phase cells (Q3) in conjunction with pH, nutrient availability, light, DIC, cell number, protein, carbohydrate, and lipid. Cultures were sampled in duplicate for each time point, total RNA extracted, and each sample sequenced via an individual lane of Illumina sequence determination. RNA-sequence analysis was used to globally monitor gene expression during nutrient-depletion and lipid accumulation under the tested growth conditions. Using TopHat and Cufflinks (see Materials and Methods), the transcript relative abundance was calculated and reported as FPKMs (Fragments Per Kilobase of exon per Million fragments mapped), a normalized quantity that is directly proportional to transcript abundance as recently reported [27-29]. To compare transcript levels between the time points, Q2 and Q3 were reported as the relative ratio compared to Q1. The Q1 time point was considered the basal transcript level condition in which cells were growing exponentially under nutrient-replete conditions with low lipid levels.

Based upon work in bacteria, transcript and protein abundances are not necessarily correlated, although some genes can show similar transcript and protein trends [30]. Therefore, different data sets (*i.e*., transcript and protein) can reveal key aspects of the physiological state of the cells. Previous studies have shown differences between transcript and protein abundances for selected genes in diatoms and yeasts [31-33] while a recent *P. tricornutum* study showed similar abundance trends [34]. The presented data are based upon transcript abundances although other mechanisms of control most likely contribute to overall metabolism.

### Global transcript differential expression

Each sample (*i.e*., biological replicate) was sequenced on a respective Illumina lane with an average of 31 million reads per sample (1 × 54 nucleotides). Cufflinks assembled 30,373 transcripts to 10,125 mapped loci, and 1,259 genes were expressed at statistically significant levels (approximately 12.5% of the genome) at all three time points ( Additional file 5: Table S2). Of all significant genes, approximately 180 genes were differentially expressed between all three time points, 546 genes between any two time points, and 177 genes between only two time points. Genes annotated as hypothetical proteins represented 37% of the significantly expressed genes (n = 465). For example, the third most abundant transcript detected in Q2/Q1 (500-fold; *p* < 0.05) and Q3/Q1 (136-fold; *p* < 0.05) is a gene of unknown function (55010) that is annotated as a pyridoxal-dependent decarboxylase. The putative *P. tricornutum* protein has significant sequence homology (44%; 6 × 10^-159^) with a coccolith-scale associated protein-1 (AB537972.1) from *Pleurochrysis carterae*. *P. carterae* has been shown to accumulate lipids [35]. While the exact role of this putative protein in *P. tricornutum* is not known, it is interesting to speculate a possible role in inorganic carbon homeostasis.

**Figure 4:**
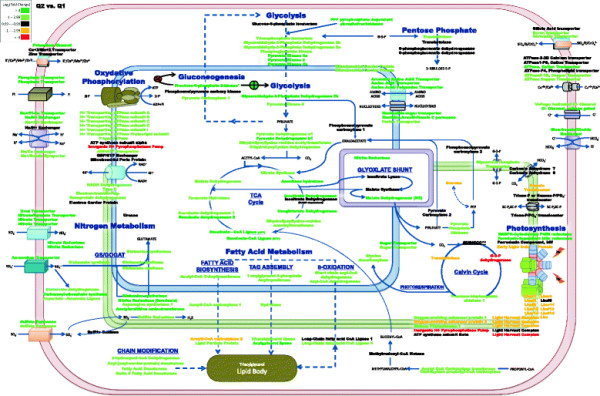
**Proposed cellular metabolic map for*P. tricornutum*during nutrient depletion and initial lipid accumulation as compared to nutrient replete conditions (Q2 vs. Q1).** Differences in fold change are based on log_2_ scale. Color scale represents up-expressed (green) and down-expressed (red) genes. Genes are represented within organelles based on predicted protein localizations (from the literature) including probable membrane bound proteins.

**Figure 5:**
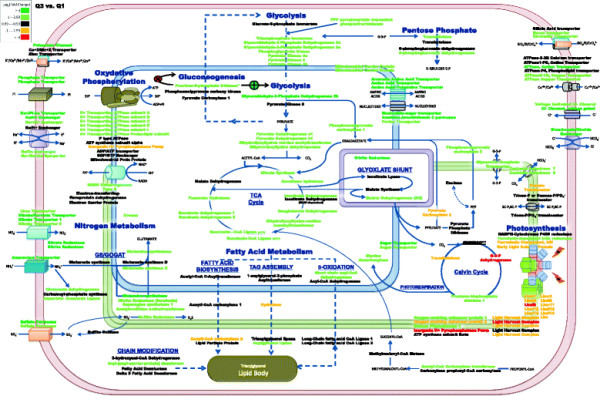
**Proposed cellular metabolic map for*P. tricornutum*during extended nutrient depletion and lipid accumulation as compared to nutrient replete conditions (Q3 vs. Q1).** Differences in fold change are based on log_2_ scale. Color scale represents up-expressed (green) and down-expressed (red) genes. Genes are represented within organelles based on predicted protein localizations (from the literature) including probable membrane bound proteins.

Approximately 170 transcripts that showed significant changes in abundance were mapped with respect to major carbon pathways and cellular compartmentalization for Q2/Q1 and Q3/Q1 and included nitrogen metabolism, oxidative phosphorylation, photosynthesis, glycolysis, TCA cycle, and fatty acid metabolism (Figures 4 and 5, respectively). A majority of the up-expressed genes during exponential-growth phase (Q2) were involved with nitrate and phosphate acquisition or utilization (*e.g.*, nitrate transporter, phosphate transporter, nitrite transporter, nitrate reductase, glutamine synthetase, and putative aspartate aminotransferase). Similar genes and/or functions remained up-expressed during transition to stationary-phase at Q3, but up-expressed genes also included a putative nucleotidase and alkaline phosphatases. The nucleotidase and alkaline phosphatases may be further responses to nutrient deprivation in order to sequester and recycle both nitrogen and phosphate. The largest down-expression at Q2 was the presumptive plastidic glyceraldehyde-3-phosphate dehydrogenase, which may coincide with a decline in photosynthetic carbon flow from 3-phosphoglycerate and phosphoglyceraldehyde phosphate to pentoses and hexoses. Light-harvesting complexes were also significantly down-expressed at Q2, and genes involved in light-harvesting complexes continued to be down-expressed into stationary-phase (Q3). These results suggest that overall energy-generation via light was down-expressed during the light cycle after exogenous N and P depletion.

### Cell-cycle

Transcript analysis revealed significant differential expression of 31 transcription factors; however, the target genes are unknown. During Q2, one transcription factor had decreased expression while all others had increased expression (2-fold to 48-fold; *p* < 0.05). During extended stationary-phase and lipid accumulation (Q3), 4 transcription factors had decreased expression and 20 transcription factors had increased expression (2-fold to 11-fold; *p* < 0.05). This also correlates to the cyclin expression and diatom specific cyclin (dsCYC) expression results. It has been proposed by Huysman *et al*. 2010 [36] that dsCYCs may act as signal integrators for a fluctuating environment (*e*.*g*., changes in light intensity, temperature, and nutrient availability). The Huysman *et al.* study reported that ds*cyc7* and ds*cyc10* were indicators of phosphate availability and that gene expression increased with increased phosphate availability. In our results, both ds*cyc*7 and ds*cyc*10 had increased expression (17-fold and 7-fold, respectively; *p* < 0.05) during Q2 when phosphate was internally available and most likely stored as polyphosphate. During Q3, ds*cyc7* and ds*cyc10* decreased in transcript abundance by 85% and 49% respectively, and coincided with the phosphate stress during Q3. Conserved cyclin (*cyc*B1) is a late-phase cell cycle (G2/M) gene that shows a slight decrease in abundance in Q2 and a nearly 4-fold increase in transcript abundance during Q3 compared to Q1. The increased expression of *cyc*B1 suggests a role during cell-cycle arrest induced by nutrient deprivation of both nitrogen and phosphorus.

### Nitrogen-limitation response

**Figure 6:**
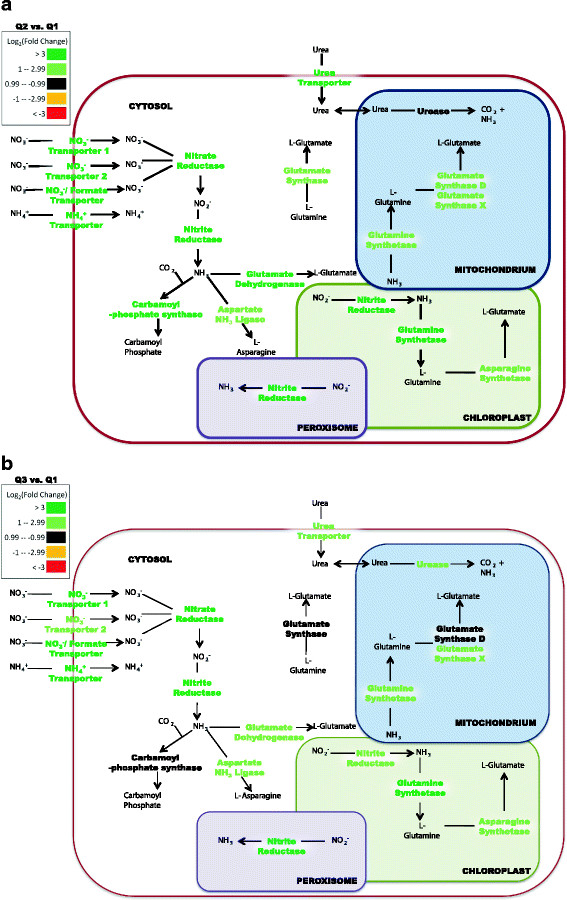
**Nitrogen metabolism gene expression of*P. tricornutum*during nutrient depletion and lipid accumulation as compared to nutrient replete conditions (Q2 vs. Q1) (A).** Nitrogen metabolism gene expression during extended nutrient depletion and lipid accumulation as compared to nutrient replete conditions (Q3 vs. Q1) **(B)**. Genes are localized to organelles based upon reported literature. Differences in fold change are based on log_2_ scale and the color scale represents up-expressed (green) and down-expressed (red) genes.

Genes involved in nitrogen metabolism were highly up-expressed at Q2 and Q3 (relative to Q1) as the cells experienced nitrate depletion and transitioned into stationary-phase (Figure 6a and 6b). The up-expression of nitrogen metabolism genes including ammonium and nitrate transporters at Q2 is most likely a result of the fast growth and biomass accumulation during exponential growth. The up-expression of transporters coincided with enzymes that utilize ammonium ions for amino acid and nucleotide pools (Figure 6a and 6b). Most of the transporters remained up-expressed in Q3, most likely as a response to continued nitrogen deprivation. However, the putative carbamoyl-phosphate synthase, glutamate synthase (cytoplasmic), and glutamate synthase (mitochondria) were no longer up-expressed in stationary-phase and the transcript levels returned to initial Q1 abundances. The elevated transcript levels for nitrate, ammonium, and urea transporters in stationary-phase suggested a cellular strategy to scavenge externally available nitrogen while the internal ammonia-utilizing enzymes were at basal transcript levels.

A recent study demonstrated that phytoplankton altered the number of transporters at the cell surface compared to internal assimilatory enzymes [37], and this result is similar to observations of nitrogen deprivation in *Chlamydomonas reinhardtii,* in which some nitrogen acquisition genes remain strongly up-expressed after nitrogen deprivation [38]. The expression pattern of carbamoyl-phosphate synthase that catalyzes carbon dioxide and ammonia condensation into carbamoyl phosphate is increased at Q2, but decreased in Q3. Carbamoyl-P synthase has been hypothesized to regulate carbon and nitrogen flow through the urea cycle in *P*. *tricornutum*[9]. The urea cycle can fix carbon into nitrogenous compounds to effectively replenish compounds needed for cellular growth, and Allen et al. (2011) postulated that the urea cycle may serve as a distribution and repackaging hub for inorganic carbon and nitrogen [39]. Although the transcript expression results do not show up-expression of the complete urea cycle, it is possible that carbamoyl-P synthase could function as a control point for the sequestration of available ammonia for anaplerotic reactions (*e.g*., pyrimidine biosynthesis) when exogenous nitrogen and DIC are low. Glutamate dehydrogenase and both glutamine synthetases remained at similar up-expressed levels during Q2 and Q3 and these results suggest that the cells expressed the GS/GOGAT (glutamine synthetase/glutamine oxoglutarate aminotransferase) pathway for ammonia sequestration possibly to recycle intracellular nitrogenous compounds (*e.g*., proteins, chlorophyll).

When exogenous nitrogen was depleted and cells entered stationary-phase, chlorophyll *a* content decreased ( Additional file 1: Figure S1) and this observation coincided with decreased gene expression for light-harvesting complex (LHC) genes (Figures 4 and 5). These observations could be explained by nitrogen-deprivation-induced chlorophyll a degradation as the cells try to maintain nitrogen allocation into stationary-phase. In Q2 and Q3, we observed an overall decrease in presumptive photosynthetic genes including chlorophyll *a* and fucoxanthin. However, most of the photosynthetic reductase components did not decrease below exponential-phase levels, and this result may suggest a basal level of light-harvesting coupled to carbon fixation even during nutrient-deplete conditions. Photosynthetic transcripts were highly up-expressed in Q1 during nutrient replete conditions, but as depletion ensued and DIC consumption was high there was a metabolic shift to reduce overall photosynthesis via a decrease in Calvin cycle genes in the chloroplast. For example, glyceraldehyde phosphate dehydrogenase (22122) was down-expressed as well as a transketolase (50819) during Q2 and Q3.

### Carbon flow

The cells encountered low DIC availability during the exponential-phase, and most, if not all, phytoplankton have evolved carbon (CO_2_)-concentration mechanisms (CCMs) to maintain photosynthetic carboxylation and reduce photorespiration via ribulose-1,5-bisphosphate carboxylase/oxygenase (RubisCO). Reinfelder [40] recently published an extensive review on CCMs in eukaryotic marine phytoplankton. It is speculated that marine diatoms have two possible types of CCMs, a biophysical and a biochemical mechanism. A biophysical CCM is the direct transport of inorganic carbon across multiple membranes using transporters and carbonic anhydrases. The other mechanism for concentrating inorganic carbon around the plastid is the use of a C4- (four carbon intermediate) CCM. In a biochemical C4-CCM, relatively high affinity carboxylases fix inorganic carbon to a C3 intermediate and form C4 compounds that can be transported to the chloroplast. The C4 intermediates are then decarboxylated to deliver the inorganic carbon to the relatively low-affinity RubisCO [41], and the C3 intermediate is recycled for another round of carbon fixation In this sense, the CCM takes advantage of the C4 carboxylases low K_m_ for inorganic carbon and affords the cell an additional mechanism to concentrate carbon. In the debate of C3 versus C4 in diatoms, evidence for both a biophysical [42-45] and biochemical CCM [34,42-49] have been presented. The *P. tricornutum* genome encodes the presumptive genes to run both C3 and C4 mechanisms, [11,46] and *P. tricornutum* is most likely using both mechanisms in response to inorganic carbon levels (discussed further below).

**Figure 7:**
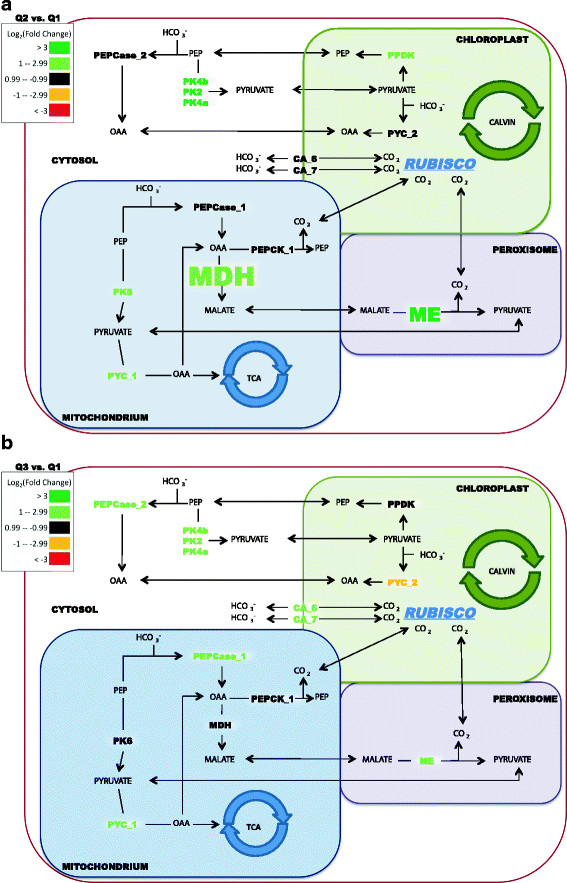
**Proposed C4 metabolism of*P. tricornutum*based on gene expression and gene localizations.** Carbon-assimilation gene expression during nutrient depletion and initial lipid accumulation as compared to nutrient replete conditions (Q2 vs. Q1) **(A)**. Carbon-assimilation gene expression during extended nutrient depletion and lipid accumulation as compared to nutrient replete conditions (Q3 vs. Q1) **(B)**. Differences in fold change are based on log_2_ scale and the color scale represents up-expressed (green) and down-expressed (red) genes. Font size is adjusted to the transcript abundances of C4 metabolism genes relative to each other.

The transcript analysis indicated a possible C4-CCM at Q2 when exogenous DIC was low, flowing through pyruvate carboxylase as the initial intermediate for carbon fixation followed by decarboxylation of malate by the malic enzyme (51970, B7FZD7) in the peroxisome (Figure 7a). In the proposed model based upon gene expression, phosphoenolpyruvate (PEP) is converted to pyruvate by pyruvate kinase_6 (mitochondria), and pyruvate is then carboxylated to oxaloacetate via pyruvate carboxylase *(pyc_*1, 30519) in the mitochondria. The oxaloacetate is converted to malate from an up-expressed (6-fold) malate dehydrogenase (*mdh*, 51297) in the mitochondria. The malate is decarboxylated by the malic enzyme (ME, 51970, B7FZD7) in the peroxisome (Figure 7a). The malic enzyme is up-expressed 8-fold, and sequence data suggest that the malic enzyme could be located in the peroxisome. Peroxisomal proteins usually have a c-terminal peroxisomal targeting signal (PTS1) peptide consensus of (S/A/C)-(K/R/H)-(L/M) [50]. The c-terminus of the malic enzyme has a SKK motif, and thus may be targeted to the peroxisome. However, an *in silico* prediction of peroxisomal genes in the *P. tricornutum* genome did not locate the malic enzyme to the peroxisome [50]; therefore, it is possible that the malic enzyme is located in the mitochondria. The malic enzyme would decarboxylate the malate to pyruvate, thus concentrating inorganic carbon that can diffuse to the plastid for fixation by RubisCO. In addition to *pyc*_1 and malic enzyme, the plastid pyruvate pyrophosphate di-kinase was up-expressed in Q2 relative to Q3 (Figure 7a and 7b). This enzyme could be involved in the moving of C3 intermediates back to the cytoplasm and/or the mitochondria for the C4-CCM. The data differ from most other proposed models in that the gene expression data suggest that the initial C3 + C1 carboxylation occurs mainly via pyruvate carboxylase_1 in the mitochondria and not through phosphoenolpyruvate carboxylase (*pepc*_1, 56026).

As the cells transitioned to stationary-phase (Q3) and the exogenous DIC increased due to decreased metabolic demand under nutrient deplete conditions, the *pyc*_1 and malic enzyme remained up-expressed relative to Q1. The pyruvate kinase_6 and the plastid *pyc*_2 were down-expressed, and the mitochondrial *pepc*_1 and cytoplasmic *pepc*_2 were up-expressed in Q3 (Figure 7b). However, the mitochondrial malate dehydrogenase was no longer up-expressed at Q3 (Figure 7b). These results suggest that C4-CCM was more active during Q2 when exogenous DIC was low due to active population growth and lipid accumulation was initiated. At Q3, C4-CCM might become less important as the cell uses *pepc*_2 to sequester bicarbonate in the cytoplasm.

Kroth *et al.* 2008 developed an annotated model for a biochemical CCM based on genome annotations of the marine diatoms, *P. tricornutum* and *Thalassiosira pseudonana*. The decarboxylating enzyme and subcellular localization were difficult to predict; however, the authors estimated the decarboxylation did not occur in the *P. tricornutum* plastid. The authors further suggested that carboxylation may occur in the mitochondria and the concentrated CO_2_ could then diffuse to the chloroplast and be fixed by RubisCO. The presented expression data is in general agreement with the predicted model, but also provides a potential route for C4-CCM based upon gene expression. Malate could be transferred into the peroxisome for decarboxylation via the malic enzyme where the CO_2_ could diffuse to the chloroplast and RubisCO.

It is not a new idea for C4 metabolism to go through PYC_1 and ME, but most models predict PEPC as the initial carboxylation step [46,47,49]. However, under CO_2_ limited-conditions in Q2 our expression data showed that *pepc*_1 is expressed at a lower level compared to *pyc*_1 (FPKM levels of 8.7 versus 102.9, respectively; *p* < 0.05). In addition, the presented data corroborate the study of McGinn and Morel (2007) that showed *P. tricornutum pepc* transcripts did not significantly increase during low CO_2_ conditions. In addition, bicarbonate addition was recently shown to increase the rate of lipid accumulation in *P. tricornutum* if added at the time of low N and low DIC [17]. The use of a C4-CCM would help explain this observation and also underscores the ability of *P. tricornutum* and similar photoautotrophs to respond to exogenous DIC for lipid accumulation.

**Figure 8:**
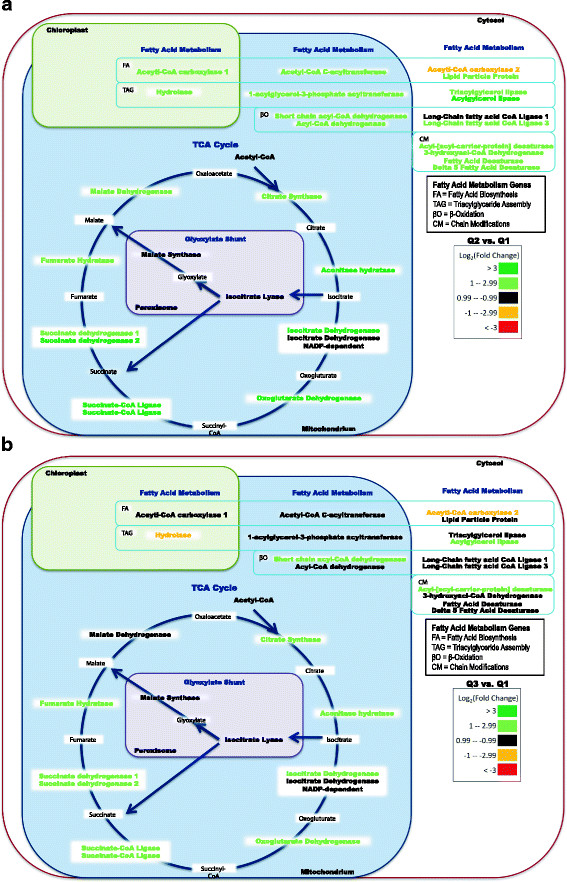
**Fatty acid metabolism, tricarboxylic acid cycle, and glyoxylate shunt related gene expression in*P. tricornutum*during nutrient depletion and initial lipid accumulation as compared to nutrient replete conditions (Q2 vs. Q1) (A).** Significant gene expression during extended nutrient depletion and lipid accumulation as compared to nutrient replete conditions (Q3 vs. Q1) **(B)**. Fatty acid metabolism genes are denoted by presumptive roles in fatty acid biosynthesis, triacylglyceride assembly, β-oxidation, and chain modifications. Differences in fold change are based on log_2_ scale and the color scale represents up-expressed (green) and down-expressed (red) genes.

As cells exited exponential-growth and entered stationary-phase, gene expression was maintained for most oxidative phosphorylation and glycolysis genes at Q2 and Q3 relative to Q1 (Figures 4 and 5). However, a majority of TCA genes declined in expression from Q2 to Q3 (malate dehydrogenase, aconitase, isocitrate dehydrogenase, oxoglutarate dehydrogenase, succinyl-CoA ligase, and succinate dehydrogenase) (Figure 4, 5 and 8). These results indicate an overall slower metabolism as the cells entered stationary-phase even though lipids continued to increase (Figure 4, 5 and 8).

The α-carbonic anhydrase_6 and 7 (CA-6 and CA-7) were up-expressed at Q3 (6.8-fold and 3.3-fold, respectively; *p* < 0.05), and these results indicated a potential role for a biophysical CCM during lipid accumulation as exogenous DIC levels increased (Figure 7b). The CA-6 and CA-7 are putatively localized in the periplastidic compartment [42,44]. As growth slowed and DIC became more available during the nutrient deplete state, C4-CCM may not have been needed, but a biophysical CCM could still increase CO_2_ flux to RubisCO by facilitating bicarbonate transport into the chloroplast. This mechanism would potentially concentrate CO_2_ in the lumen surrounding the plastid for increased delivery to RubisCO [46]. In addition, CA-6 and CA-7 in the periplastidic compartment could provide a pressure barrier for CO_2_ efflux from the plastid [43]. The increase in *pepc*_1 and *pepc*_2 transcript levels during Q3 may represent potential roles as anaplerotic enzymes to maintain TCA cycle intermediates and overall cellular maintenance. A presumptive Cl^-^/HCO_3_^-^ exchanger gene (54405) was up-expressed (22-fold; *p* < 0.05) at Q2 when exogenous DIC was low. At Q3, the gene expression for 54405 decreased back towards basal levels as exogenous DIC levels increased during stationary-phase, and these combined results could indicate a combined biophysical and biochemical strategy of CCM at Q2.

### Fatty acid metabolism

Understanding how carbon is partitioned as nutrient conditions change is important to predict carbon flow for lipid accumulation. Previous work has shown that stress caused by low availability of nitrogen can increase lipid content in different microalgae [23,51-53], and we observed a similar response in *P. tricornutum* (Figures 2 and 3). However, from a genome-wide expression standpoint, algal TAG accumulation is not well studied. Hu *et al*. [16] reviewed *de novo* fatty acid biosynthesis and TAG biosynthesis in microalgae, and proposed that a significant portion of TAGs are made from recycling of phosphoglycerides and glycolipids instead of *de novo* fatty acid biosynthesis *per se*. In fact, the activity and up-expression of acetyl-CoA carboxylase was a focus for previous studies, including the U.S. Department of Energy’s Aquatic Species Program [15].

At Q2, as exogenous nitrate and phosphate were depleted, the plastid acetyl-CoA carboxylase_1 and hydrolase were up-expressed (approximately 2.5 to 3.0-fold; *p* < 0.05) compared to Q1 (Figure 8a). In the mitochondria, the acetyl-CoA acyltransferase (28068) and the acylglycerol acyltransferase (45551) genes were up-expressed (2.5-fold; *p* < 0.05) at Q2/Q1. Gene expression for the same genes declined in Q3 (*i.e*., the levels were similar to Q1). The acetyl-CoA carboxylase_2 (55209) was down-expressed (approximately 2-fold; *p* < 0.05) and remained at similar levels at Q3 (Figure 8b). A gene annotated as a lipid particle protein (45518) was up-expressed at Q2, but expression declined at Q3 (Figure 8). These results suggest the plastid acetyl-CoA carboxylase showed greater expression changes (3-fold) than the cytoplasmic acetyl-CoA carboxylase, but neither was strongly up-expressed at the transcriptional level during prolonged lipid accumulation (*i.e*., Q3). Similar results were recently reported when RNA-seq was used to evaluate gene expression in *Chlamydomonas reinhardtii*, in which only modest changes in gene expression of fatty acid metabolism genes were reported [37].

The results demonstrate modest increases in transcripts involved in fatty acid biosynthesis, TAG assembly, fatty acid chain modifications, and β-oxidation at Q2, but an overall return to early-exponential abundances at Q3 during an extended time period of lipid accumulation (Figure 8). During the initial increase in lipid accumulation (Q2), a 3-fold increase in acetyl-CoA carboxylase_1 (*acc_*1, plastid) was observed. This is an expected result based on the fact that ACC is the enzyme involved in the committed step for fatty acid biosynthesis. In addition, 1-acylglycerol-3-phosphate acyltransferase (*agat*) which is the second step in TAG formation was up-expressed 6-fold at Q2 (Figures 4 and 8). Genes involved in β-oxidation and fatty acid chain modifications were also up-expressed at Q2, and these changes may be in response to changing membrane dynamics as well as recycling of nitrogen and phosphorus associated with different lipid classes (Figure 8a). For example, an acyl-ACP desaturase was up-expressed 7.4-fold and 3.5-fold in Q2 and Q3, respectively and may be involved in incorporation and/or production of unsaturated fatty acids. However, even though lipid accumulation was at a faster rate (specific NR fluorescence/t) after Q2, most of the genes associated with fatty acid biosynthesis returned to basal levels (Q1), including *acc_*1 and *agat* (Figure 8).

During prolonged lipid accumulation (Q3), the direct fixation of C3 carbon might play a more significant role for overall carbon flow towards TAG accumulation (*i.e*., higher exogenous DIC and lower expression of C4-related genes). Our results suggest that the build-up of precursors to the acetyl-CoA carboxylases (*i.e*., acetyl-CoA) may be a more significant contribution toward TAG accumulation than the actual enzyme levels of acetyl-CoA carboxylases *per se*. In addition, there could be alternative routes of carbon intermediates towards overall TAG accumulation. For example, *P*. *tricornutum* has a putative methylmalonyl-CoA mutase gene that was slightly up-expressed in Q2 and Q3 (1.3 and 2.0-fold, respectively; *p* < 0.05). The putative methylmalonyl-CoA mutase (51830) might convert succinyl-CoA to methylmalonyl-CoA, and such a conversion could link TCA intermediates to fatty acid biosynthesis. In addition, a putative propionyl-CoA carboxylase gene (51245) that could carboxylate propionyl-CoA via bicarbonate to produce methylmalonyl-CoA was up-expressed 2-fold at Q2 and 1.8-fold at Q3 (*p* < 0.05). These results suggested alternative routes for carbon fixation in addition to C3 and C4 intermediates.

## Conclusions

*P. tricornutum* has evolved to survive the varying and often nutrient-limiting conditions encountered in marine habitats. In response to nitrogen and exogenous phosphate depletion, cells maintained up-expression of transcripts for nitrogen transport and assimilatory genes as well as presumptive phosphate transporters. Under the growth conditions tested, cell numbers continued to increase after the depletion of exogenous phosphate, and these results corroborated the notion that *P. tricornutum* can store internal phosphate [54]. The extracellular phosphate was depleted before nitrate, and Terry *et al*. (1985) [55] surmised that the ability of *P. tricornutum* to accumulate nitrogen was extremely restricted during phosphate limitation. Lipids started to accumulate when exogenous phosphate was approximately half the initial levels and the external N:P ratio was approximately 27. The rate of lipid accumulation (specific NR fluorescence/time) increased (3.3-fold) once external nitrate was depleted. These results indicate that phosphate depletion could be an initial trigger for lipid accumulation that was “magnified” upon nitrate depletion in *P. tricornutum*.

The lipid accumulation at Q2 coincided with up-expression for acetyl-CoA carboxylase (*acc*_1) and 1-acylglycerol-3-phosphate acyltransferase. At Q2, *acc*_1 was up-expressed 3.1-fold, the *acc* β-subunit was up-expressed 3.7-fold, and the acyltransferase was up-expressed 5.8-fold. However, the expression for these genes declined during Q3 when lipid was being accumulated at a faster rate (specific NR fluorescence/time). In addition, *acc*_2 was down-expressed 2-fold at Q2 and 5-fold at Q3. These results could suggest that carbon is being “pushed” into fatty acid synthesis via elevated acetyl-CoA and NADPH levels and thus not being “pulled” by a large abundance of fatty acid synthesis activity. While it is possible that protein levels are increased for fatty acid biosynthesis, the transcript data do not suggest this scenario.

A “push” condition is when flux processes occur prior to a pivot point (“hinge”), and a “pull” condition occurs following the “hinge” [56]. With respect to lipid accumulation, the “hinge” is the committed step in fatty acid biosynthesis (*i.e*., acetyl-CoA carboxylase), and we postulate that a “push” condition accounts for a significant portion of the metabolic flow based upon gene expression. If acetyl-CoA was being pulled, up-expression would be expected to be maintained throughout lipid accumulation (Q2 through Q3) for most fatty acid and TAG biosynthetic genes. This metabolic step has been tested by overexpression of *acc*[7,15], but overexpression did not result in an increase of overall lipid biosynthesis.

We conclude that during the transition from growth to lipid accumulation, *P. tricornutum* utilized pyruvate carboxylase in the mitochondria as the primary inorganic fixation step in a C4 pathway with subsequent decarboxylation of malate by the malic enzyme in the peroxisome to concentrate CO_2_ for diffusion to the chloroplast. During the initial transition, exogenous DIC was low, and therefore, it appeared that a biochemical mechanism to concentrate carbon predominated. At the later time point (Q3), DIC levels had returned to initial levels, lipid accumulation continued, but two carbonic anhydrases (CA_6 and CA_7) were up-expressed. These results indicate that a biophysical carbon-concentrating mechanism then predominated to maintain carbon flow for CO_2_ reduction during nutrient deprivation.

The cells appeared to continue CO_2_ reduction when population growth was arrested and different carbon-concentrating mechanisms were used dependent upon exogenous DIC levels. The fixed carbon as C3 and/or C4 intermediates can then be “pushed” into fatty acid biosynthesis. Alternative pathways that could utilize HCO_3_^-^ were also suggested by the gene expression profiles (*e.g*., propionyl-CoA and methylmalonyl-CoA). In addition, a putative decarboxylase with sequence homology to a coccolith-associated protein was significantly up-expressed. Coccoliths are external, CaCO_3_ plates formed by some single-celled algae (coccolithophores) [57], and the presumptive *P. tricornutum* protein could be involved in carbon homeostasis. Further work is needed (proteomic, metabolomic, modeling) to provide more insight into metabolic control points that are crucial for the routing of fixed carbon into lipid accumulation by photoautotrophs, but our results based upon genome expression analysis suggested multiple routes for inorganic carbon flow during lipid accumulation. Future work includes proteomic analyses for the identification of key proteins and enzymes for lipid accumulation and storage, as well as metabolomic analysis, particularly in response to dissolved carbon. It will be advantageous to consider the type and level of inorganic carbon for bio-oil production systems and how different microalgae respond during autotrophic, lipid accumulation. With respect to biofuel production, an improved understanding of how carbon is potentially “pushed” into fatty acid biosynthesis will allow for the development of metabolic networks that can be engineered to maximize inorganic carbon flow into TAG production.

## Methods

### Culture and growth conditions

*Phaeodactylum tricornutum* (Pt1) Bohlin Strain 8.6 CCMP2561 (Culture Collection of Marine Phytoplankton, now known as NCMA: National Center for Marine Algae and Microbiota) was used in all experiments. Characterization of the *P. tricornutum* strain 8.6 (Pt1) and its relationship to other *P. tricornutum* strains was previously described [10]. Pt1 was grown at 20  °C in 1.25 L photobioreactors in ASPII media as previously described [21,22], and the medium was buffered at approximately pH 8.0 with Tris-base. Cells were grown on a 14:10 light (450 μE^-1^sec^-1^):dark cycle at 20  °C. Tubes were aerated with ambient air at a regulated flow rate of 0.40 L min^-1^ and CO_2_ from air was the only available source of carbon for the diatoms. Initial inocula were grown in similar growth conditions and after two transfers were inoculated into photobioreactors during exponential-phase to a cell concentration of approximately 1x10^5^ cells x mL^-1^. Contamination checks were performed using ASPII medium supplemented with 0.5% yeast extract and 0.5% glucose as well as plating on R2A agar. Cultures remained axenic throughout experiments.

### Physiological parameter analysis

Cells and media were collected and analyzed daily to measure chlorophyll *a,* pH, dissolved inorganic carbon (DIC), nitrate, phosphate, protein, carbohydrate, cell number, and Nile Red fluorescence. All measurements were done in triplicate. Cells (1 mL) were collected daily via centrifugation (10,625 × g, 10 min) at 4 °C. The supernatant was removed and stored for nitrate and phosphate analysis at 4 °C. For chlorophyll *a* extraction, 100% methanol (1 mL) was added to the pellets followed by 30 seconds of mixing to extract chlorophyll *a,* and extracts were stored in the dark at 4 °C for 24 hours. Cellular debris was pelleted (10,625 × g, 10 min) and the methanol extract was measured on a spectrophotometer. Absorbance was measured at 652 nm and 665 nm and concentrations (μg/mL) calculated using equations reviewed by Porra (2002) [58]. Absorbance was measured on a spectrophotometer (UV-1700 PharmaSpec UV–vis, Shimadzu). The medium pH was measured using a standard tabletop pH meter. Daily, cell culture (7 mL) was filtered (0.2 μm nylon membrane filters) in preparation for DIC measurement using a Formacs^HT^ TOC/TN Analyzer (SKALAR, Buford, GA). The analysis of nitrate and phosphate were done by following the manufacturer’s colorimetric assay protocols Szechrome NAS (Polysciences, Inc.) and SensoLyte MG Phosphate Assay Kit (AnaSpec), respectively. The absorbance measurements were taken on a 96-well plate spectrophotometer (Synergy HT, Biotek). Protein was extracted by boiling 1 mL of pelleted (10,625 × g, 10 min) cells in 100 mM (Tris–HCl, 4% SDS, at pH 6.8) for 5 min [59]. Proteins were precipitated overnight at 4 °C in 10% TCA/acetone. Precipitated protein was pelleted at 4 °C and supernatant was removed. Proteins were then washed with 100% acetone and re-pelleted. Supernatant was decanted and precipitated proteins were rehydrated in Tris–HCl. Protein concentration was measured by a Qubit Protein Assay and the Qubit Fluorometer (Invitrogen). The analysis of carbohydrates was performed by following a modified monosaccharide protocol previously described by Chaplin et. al (1994) [60]. Cells (1 mL) were pelleted and the supernatant decanted, and L-cysteine HCl (1 ml; 700 mg/L) in cold sulfuric acid was added to cells, vortexed, and boiled at 100°C for 3 minutes. Samples were rapidly cooled on ice and absorbance measured at 415 nm with a 96-well plate spectrophotometer (Synergy HT, Biotek).

For lipid accumulation, we followed the Nile Red assay protocol as previously described by Cooksey et al. [24]. Cells were diluted with 1.8% NaCl (to minimize cell lysis) to an appropriate cell density that correlated to a linear relationship of cell density and Nile Red florescence that can be measured spectrofluorometrically. Nile Red (Sigma-Aldrich) solution (4 μL of 250 μg/mL in acetone) was added to a cell suspension (1 mL). Specific fluorescence was determined by dividing the Nile Red fluorescence intensity by the cell number. Cell density was monitored via cell counts in duplicate with a hemocytometer.

### RNA sampling, extraction, and sequencing

**Figure 9:**
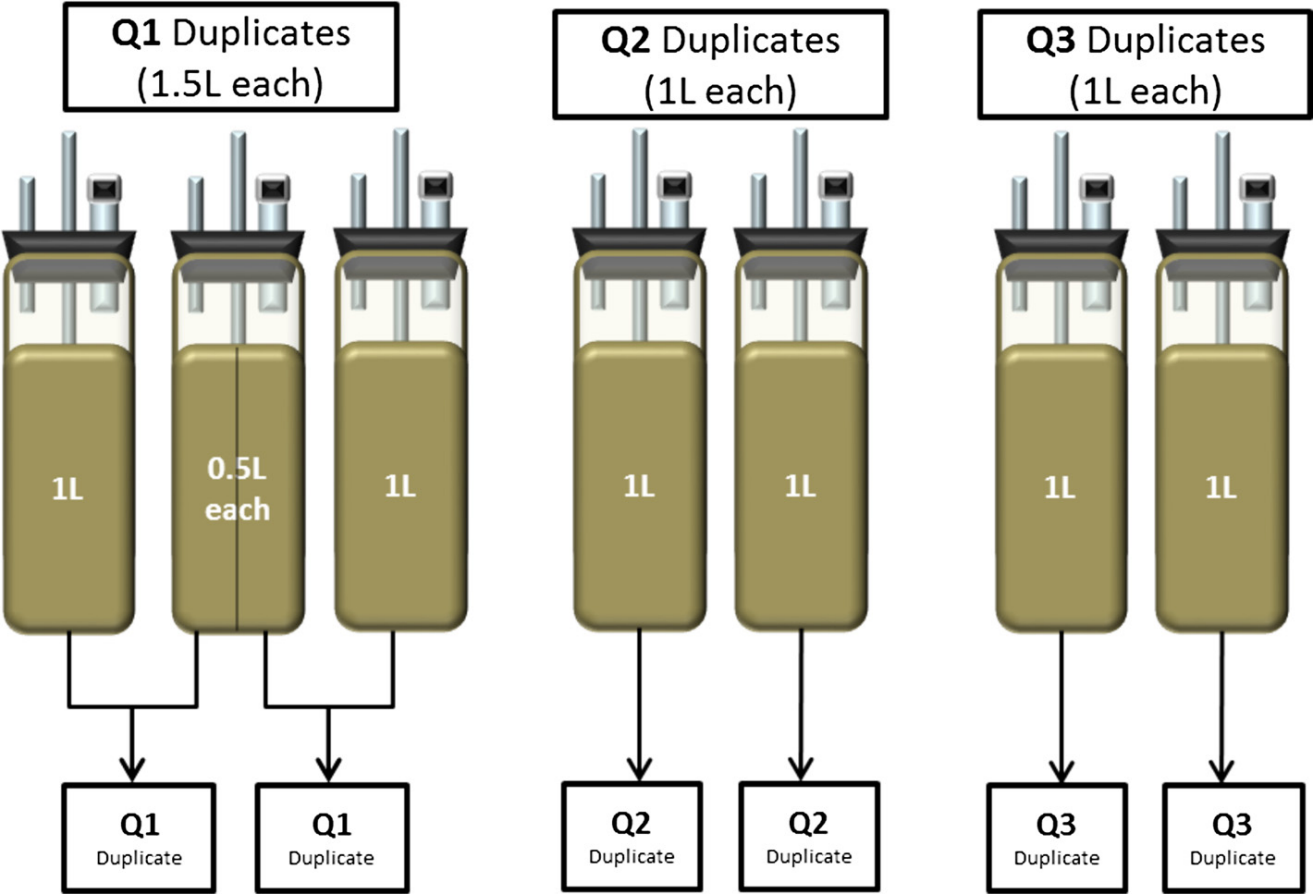
Schematic representation of temporal biomass sampling from replicate bioreactors.

RNA extraction was done in duplicate from seven identical photobioreactors. At early exponential phase (Q1), 1.5 L of culture biomass (1 L from 1 reactor plus 0.5 L from a replicate reactor) were collected (10,625 x g, 10 min at 4 °C) for each duplicate, the supernatant was discarded, and the collected cells were immediately flash frozen in liquid nitrogen (Figure 9). The use of three reactors for duplicates were necessary due to the lower cell density in the early exponential-phase. The same collection procedure was used for late exponential (Q2) and stationary phases (Q3) when cell numbers were higher except that 1 L was collected for each in duplicate. Flash frozen aliquots were stored at −80 °C until RNA extraction. Total RNA was extracted following the Spectrum Total RNA kit (Sigma Aldrich-STRN50) protocol with on-column DNAase digestion using DNase10 (Sigma-Aldrich) as suggested by the manufacturer. Initial lysis was done by a 30 second cell disruption via sonication. The protocol was performed in an RNAse free-hood to prevent RNA degradation. Total RNA was not pooled but kept as respective duplicates, and samples were sent to the National Center for Genome Resources (NCGR, Santa Fe, NM). At NCGR, mRNA was isolated twice by polyadenylation purification, and first and second strand DNA was generated with random primers. Gel products were purified and PCR enriched. Products were quantified on a Nanodrop fluorometer (Thermo Scientific) and checked for quality on a Bioanalyzer 2100 (Agilent). Using Illumina’s standard protocols, flow cell construction and sequence determination was performed. Each duplicate was run on a single lane (6 total samples, 3 duplicates). Single reads were put into FASTQ format for analysis.

### Transcript assembly

Single-end Illumina reads were analyzed using the following pipeline. First, reads from each sample were aligned against the *P. tricornutum* genome (v. 8) from ENSEMBL [61] with the TopHat algorithm [62]. TopHat was configured to use known splice junctions for Pt1, as retrieved from genome annotation version 61 from ENSEMBL. Mapped reads were assembled into putative transcripts using Cufflinks [27]. Abundance of each transcript was estimated in each sample then compared across samples using Cuffcompare and Cuffdiff, parts of the Cufflinks toolkit ( Additional file 5: Table S2) [27]. Similar pipelines have been validated as well as the accuracy of RNA-seq with spike in experiments and qPCR comparisons [28,29,63-66].

### Transcript identification and pathway analysis

Cufflinks output files had transcripts identified by uniprot accessions. Using the DAVID (Database for Annotation, Visualization, and Integrated Discovery) [67] Gene ID conversion tool, uniprot accessions were converted to Locus Tag IDs and Protein IDs. Once all accessions were converted, the DAVID Functional Annotation tool was used to retrieve gene names as well as KEGG (Kyoto Encyclopedia of Genes and Genomes) Pathway information. For genes that were identified as hypothetical proteins, searches were performed on the JGI *Phaeodactylum tricornutum* v2.0 genome website (http://genome.jgi-psf.org/Phatr2/Phatr2.home.html) and based on best hits, %ID, score, and consistency of the top hits, genes were either identified or remained as hypothetical. Genes were also searched on NCBI and ENSEMBL genome browsers for cross-referencing. To assign genes into pathways, we used KEGG maps for *P. tricornutum* as a backbone. Genes for major pathways were searched manually to find genes not directly annotated in the *P. tricornutum* KEGG maps. Gene lists were compiled for the major pathways and developed into network maps.

Organelle targeting for transcript products was done based on annotations from the databases of JGI, NCBI, and ENSEMBL. If no localization was found, eukaryotic organelle localizations were predicted with TargetP 1.1. server [68] in both plant and non-plant mode. Amino acid sequences were also checked for a peroxisomal targeting sequence (SKL, serine-lysine-leucine). If potential targeting was not identified, we assumed that the gene product occurred in the cytoplasm. If the gene was an integral membrane protein we again checked JGI, NCBI, and ENSEMBL, and if targeting could not be determined we located the gene in the most biologically relevant membrane (*e.g*., light harvesting complex in the plastid).

### Elemental analysis

To better understand the effects of nutrient limitation on lipid accumulation, the elemental carbon:nitrogen:phosphorus (C:N:P) composition was determined at Q1, Q2, and Q3. Cells were grown in the same conditions and harvested at the same time points at which RNAseq occurred. Cells were collected via centrifugation (10,625 x g, 10 min at 4°C). Supernatant was discarded and concentrated cells were transferred to 50 mL tubes and centrifuged (10,625 x g, 15 minutes at 4°C). Supernatant was discarded and pelleted cells were placed in a 60°C incubator to dry. Dried cells were sent to the University of Missouri Soil Testing and Plant Diagnostic Service Laboratories (Columbia, MO) for elemental analysis. Percentages of carbon, nitrogen, and phosphorus were used to calculate the C:N:P ratios in the cells.

## Abbreviations

TAG, Triacylglyceride; NR, Nile Red; DIC, Dissolved Inorganic Carbon; CYC, Cyclin; CCAP, Culture Collection of Algae and Protozoa; CCMP, Formerly Culture Collection for Marine Phytoplankton, now NCMA, National Center for Marine Algae and Microbiota; Pt1, Phaeodactylum tricornutum strain 1; EST, Expressed Sequence Tag, RNA-seq, RNA-sequencing; FPKM, Fragments Per Kilobase of exon per Million reads; dsCYC, diatom specific cyclin; GS/GOGAT, Glutamine synthetase/glutamine oxoglutarate aminotransferase; LHC, Light harvesting complex; CCM, Carbon concentrating mechanism; C3, 3-carbon metabolite; C4, 4-carbon metabolite; PEP, Phosphoenolpyruvate; PYC, Pyruvate carboxylase; MDH, Malate dehydrogenase; ME, Malic Enzyme; PTS1, Peroxisomal targeting signal; pepc, phosphoenolpyruvate carboxylase; RubisCO, Ribulose-1, 5-Bisphospate carboxylase oxygenase; TCA, Tri-carboxylic cycle; CA, Carbonic anhydrase; ACC, Acetyl-CoA carboxylase; ACP, Aceyl carrier protein; NADPH, Reduced form of Nicotinamide adenine dinucleotide phosphate; ASPII, Aquatic Species Program; TCA/Acetone, Trichloroacetic acid in acetone; NCGR, Nation Center for Genome Resources; DAVID, Database for Annotation, Visulization, and Integrated Discover; KEGG, Kyoto Encyclopedia of Genes and Genomes; SKL, Serine-lysine-leucine.

## Competing interests

The authors declare that there are no competing interests.

## Authors’ contributions

JV carried out growth, biomass harvest, activity assays, RNA extraction, participated in RNA-Seq data analysis, and drafted the manuscript. AM assisted in RNA-Seq data analysis and statistics. RPC, RG, KEC, BMP, and MWF participated in study design, study coordination, data analysis/interpretation, and manuscript preparation. All authors read and approved the final manuscript.

## Supplementary Material

Additional file 1 **Figure S1.***P. tricornutum* growth curve (▲) showing nitrate (○) depletion and the chlorophyll a (♦) content. Click here for file

Additional file 2 **Table S1.** Elemental analysis of *P. tricornutum* (carbon, nitrogen, and phosphorus) at the three time points (Q1, Q2, and Q3) during nutrient depletion and subsequent lipid accumulation. Values represent averages of duplicates. Click here for file

Additional file 3 **Figure S2.** Specific carbohydrate (μg/cell) during growth of *P. tricornutum.* Click here for file

Additional file 4 **Figure S3.** Color image of *P. tricornutum* stained with Nile Red. The image was taken with an Infinity 2 color camera (Lumera Corporation) at 600x-magnification using a Nikon Eclipse E800 epifluorescent microscope with a B-2A filter. Click here for file

Additional file 5 **Table S2.** Tabulated data of FPKMs, confidence intervals, fold ratio, and Log_2_ fold ratios for Q1, Q2, and Q3 genes (protein IDs) for genes with significantly changed gene expression. Click here for file
