# Supplementary figures and images for: Potential role of multiple carbon fixation pathways during lipid accumulation in Phaeodactylum tricornutum
Source: Biotechnol Biofuels. 2012 Jun 6;5:40. doi: 10.1186/1754-6834-5-40 (PMC3457861; doi:10.1186/1754-6834-5-40)

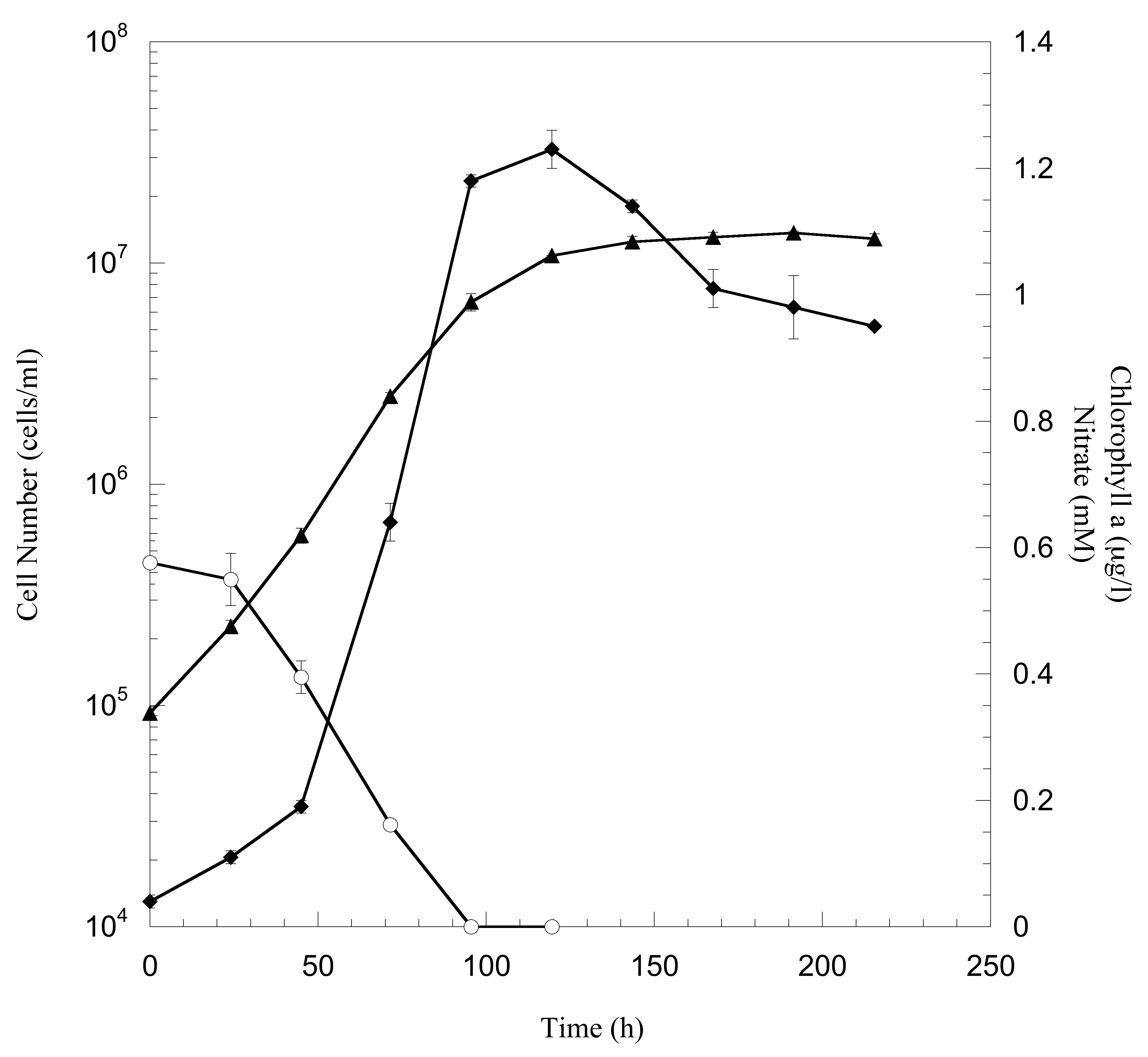

Supplement: Additional file 1 — Figure S1.P. tricornutum growth curve (▲) showing nitrate (○) depletion and the chlorophyll a (♦) content. [file 1754-6834-5-40-S1.tiff]

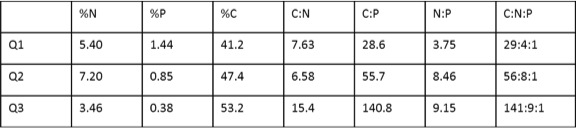

Supplement: Additional file 2 — Table S1. Elemental analysis of P. tricornutum (carbon, nitrogen, and phosphorus) at the three time points (Q1, Q2, and Q3) during nutrient depletion and subsequent lipid accumulation. Values represent averages of duplicates. [file 1754-6834-5-40-S2.jpeg]

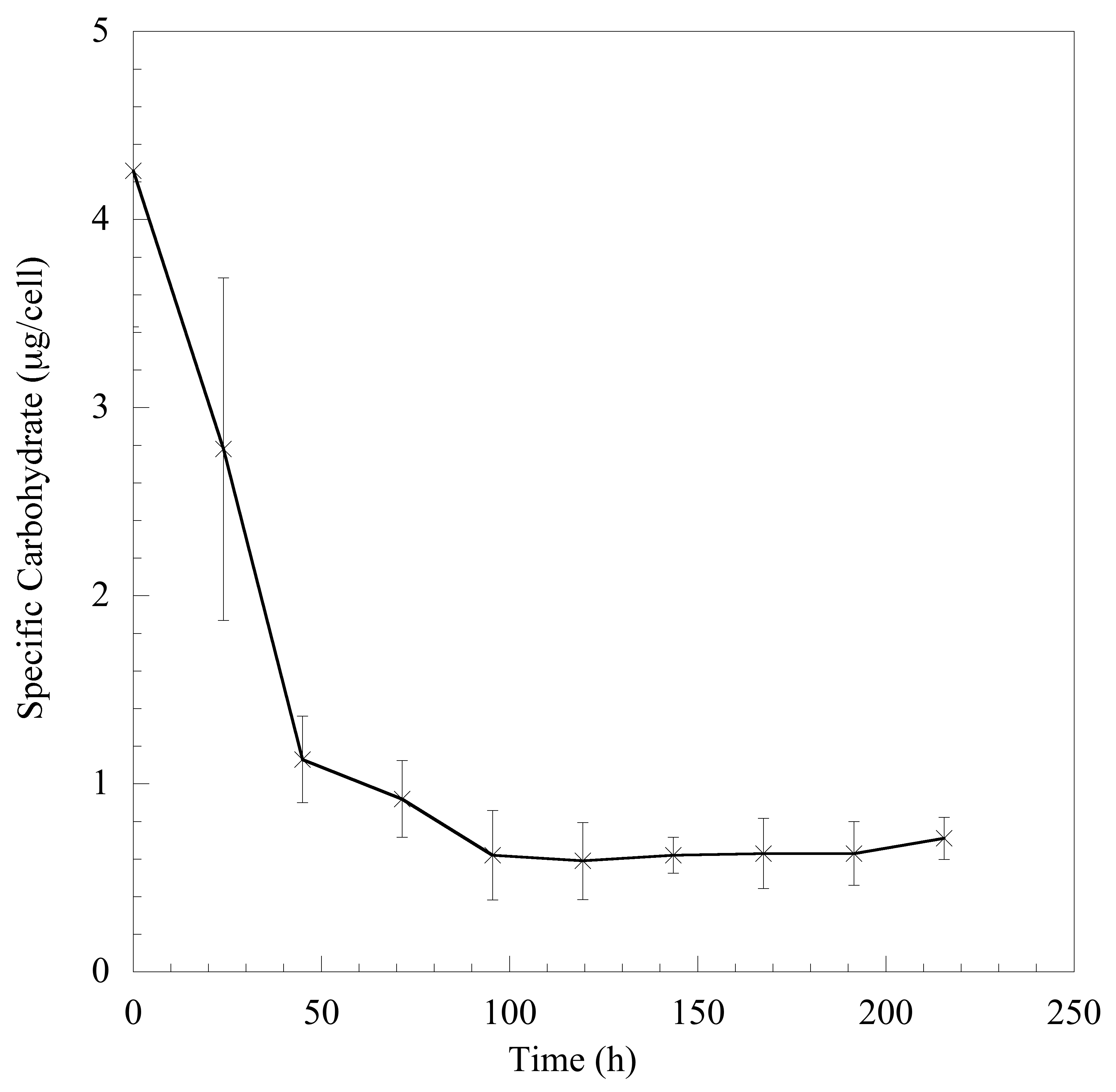

Supplement: Additional file 3 — Figure S2. Specific carbohydrate (μg/cell) during growth of P. tricornutum. [file 1754-6834-5-40-S3.tiff]

## Slide 1
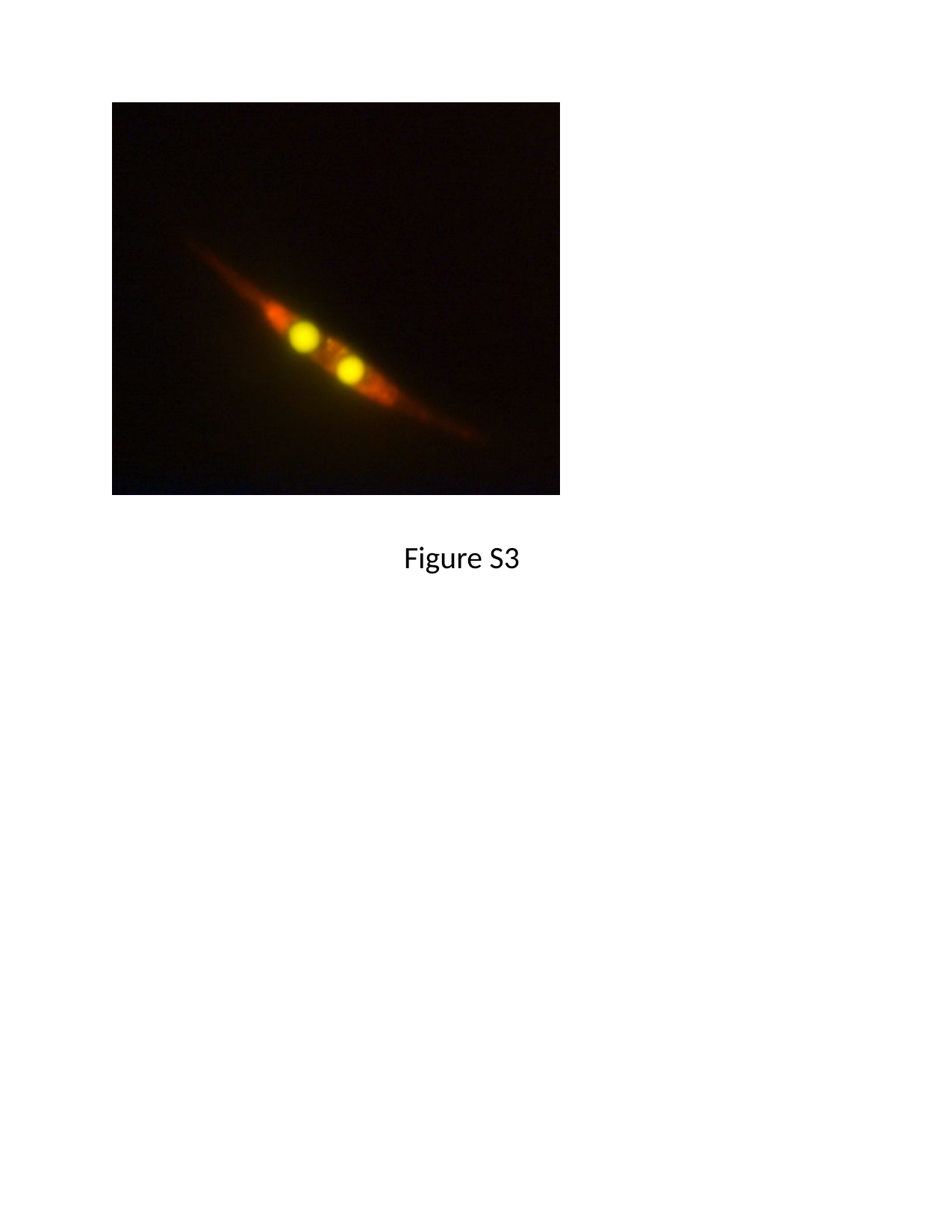

Figure S3

Supplement: Additional file 4 — Figure S3. Color image of P. tricornutum stained with Nile Red. The image was taken with an Infinity 2 color camera (Lumera Corporation) at 600x-magnification using a Nikon Eclipse E800 epifluorescent microscope with a B-2A filter. [file 1754-6834-5-40-S4.ppt]
